# “No Chit Chat!” A Warning From a Physical *Versus* Virtual Robot Invigilator: Which Matters Most?

**DOI:** 10.3389/frobt.2022.908013

**Published:** 2022-07-22

**Authors:** Muneeb I. Ahmad, Reem Refik

**Affiliations:** Department of Computer Science, Swansea University, Swansea, United Kingdom

**Keywords:** invigilation, human–robot interaction, embodiment, virtual agent invigilator, robot invigilator, educational robotics

## Abstract

Past work has not considered social robots as proctors or monitors to prevent cheating or maintain discipline in the context of exam invigilation with adults. Further, we do not see an investigation into the role of invigilation for the robot presented in two different embodiments (physical vs. virtual). We demonstrate a system that enables a robot (physical and virtual) to act as an invigilator and deploy an exam setup with two participants completing a programming task. We conducted two studies (an online video-based survey and an in-person evaluation) to understand participants’ perceptions of the invigilator robot presented in two different embodiments. Additionally, we investigated whether participants showed cheating behaviours in one condition more than the other. The findings showed that participants’ ratings did not differ significantly. Further, participants were more talkative in the virtual robot condition compared to the physical robot condition. These findings are promising and call for further research into the invigilation role of social robots in more subtle and complex exam-like settings.

## 1 Introduction

Academic dishonesty (cheating) is a globally infamous phenomenon. It takes many shapes and forms, and as the years go by, students become more creative with their cheating methods. Cheating has been viewed as a “cognitive shortcut” that reduces the reliability of test results to assess academic gain (Anderman and Murdock, 2007). Cheating has serious implications. For instance, nursing students who cheat during academic years have a higher probability of falsifying clinical data later on in their careers (Park et al., 2013). Proctoring is one of the strategies used to prevent cheating. However, proctoring is not very cost-effective, as the reported expenses by the British Council for invigilators alone were close to *£*67M in 2017, *£*74M in 2018 (Council, 2019), and *£*77M in 2019 (Council, 2020). The significant social, economic, and moral implications of cheating are enough to present a case to develop novel and efficient solutions to prevent cheating.

Social robots have a long history of use in the educational landscape. The role has transformed from passive (as a tool) to active (as a peer, tutor, or tutee) (Alves-Oliveira et al., 2016; Belpaeme et al., 2018). Recent reviews published in the human–robot interaction (HRI) literature showed that the role of an invigilator for a social robot received limited attention (Mubin et al., 2013; Ahmad et al., 2017; Belpaeme et al., 2018; Rosanda and Starcic, 2019). With the ongoing pandemic circumstances, we understand that an invigilator role for the robot can have inevitable advantages. We further see a future where proctoring could be a collaboration between humans and robots. Robots are useful for repetitive and mechanical tasks. Hence, robots monitoring an exam hall could be cost-effective and help reduce human effort.

A few studies have explored the impact of a robot on academic dishonesty displayed by adults, where the robot had the role of an invigilator (Hoffman et al., 2015; Petisca et al., 2020; Ahmad et al., 2021). Another study focused on young children and compared the presence of a robot and a human as an invigilator (Mubin et al., 2020). However, these studies present several limitations, including the passive role of the robot, the use of artificial tasks and environments, and small sample sizes. A passive invigilator robot is not reactive and does not respond to user behaviours. An active invigilator robot adapts to users’ verbal and non-verbal behaviours.

We also see a body of research that has attempted to highlight the benefits of physical embodiments of the social robot over virtual ones in various settings (Deng et al., 2019). To the best of our knowledge, the fundamental question of whether an active physical robot invigilator would be preferred over an active virtual robot invigilator has not been studied, particularly in the context of a robot invigilator that can recognise cheating or indiscipline at a minimum level and react accordingly.

Considering these gaps, we investigate the following research questions (RQs). RQ1: can an active physical robot invigilator prevent cheating more effectively than an active virtual robot invigilator? RQ2: does an examinee prefer a physical robot invigilator over a virtual robot invigilator? RQ3: would an examinee indulge in indiscipline more with a virtual robot invigilator rather than a physical robot invigilator? RQ4: do examinees respond to the warnings given by a physical robot invigilator over a virtual robot invigilator?

To investigate these questions, we programmed the Nao robot (physical and virtual) to detect two behaviours suggesting cheating or indiscipline in an exam setting: 1) looking towards another examinee’s paper and 2) speaking during the exam setting. Once detected, the Nao robot was capable of warning examinees. We conducted two different studies to investigate our RQs, and our contributions are as follows:

• We demonstrate a fully autonomous system that enables a robot (physical or virtual) to monitor examinees in an exam setting. Further, we have made our code available for reuse.
• We conducted an in-person and an online evaluation of the system to understand participants’ perception of a physical and virtual robotic invigilator in terms of quality of invigilation, trust, and avoiding cheating or indiscipline in an exam-like environment.
• We show that participants preferred the physical robot invigilator over the virtual robot in an online study. However, no significant preference difference was found in the in-person evaluation.
• We show that participants were significantly more talkative in the presence of the virtual invigilator robot.
• Interestingly, we show a positive relationship between the physical robot invigilator’s warnings and participants’ peeking behaviour. Conversely, we show a positive relationship between the virtual robot invigilator’s warnings and participants’ speaking behaviour.

The rest of this study describes related work, the study design, experimental setup, and results. We discuss the significance of the findings in the context of previous work, the limitations of this study, and directions for future research. Finally, we conclude with a summary of our findings.

## 2 Background

### 2.1 Social Robots in Education

Social robots have played the role of a tutor or a peer in the education domain. Further, they proved to be effective in raising both intellectual and emotional gains in the following roles. A tutor robot assists pupils by helping them find the correct answers, providing mini-tutorials, or even just supervising them (Belpaeme et al., 2018). In regard to the role of the robot in education, Alves-Oliveira et al. studied the way students assigned roles to an educational robot by asking them to fill a survey and choosing the role they saw as most fitting out of eight options. Despite clearly introducing the robot as a teacher, a very small percentage chose the role of tutor. The majority of the students chose the role of a friend or classmate. This study illustrates that the role perception of a robot is not as straightforward as it seems (Alves-Oliveira et al., 2016).

It is argued that education is directed not only at learning technical skills but also at moral stability through creating values such as integrity, loyalty, and discipline (Rahim and Rahiem, 2012). These values are often reflected by the student’s degree of reverence and appreciation for the agent playing various roles in an education setting. This role can be a mentor, advisor, invigilator, or monitor in addition to a teacher. In particular, the role of an invigilator for social robots is under study. A few studies have explored the role of the invigilator in the context of the impact of a robot’s presence on people’s honesty with regard to cheating (Hoffman et al., 2015; Petisca et al., 2020).

Mubin et al. described the possibility of a robot relieving human teachers completely of their role as unrealistic and undesirable (Mubin et al., 2020). They suggest that education not only pertains to academic skills but also includes learning morals and ethics. Hence, their study investigated the effectiveness of a robot as an invigilator during exams with young children. Invigilators are meant to monitor students during an exam and prevent any dishonest behaviour. From their experiments, they concluded that the Nao robot could prevent cheating but not indiscipline, where there was much talking amongst the students but no cheating. However, this might have been an exciting reaction to the novelty of a robot invigilator. Petisca et al. (2022) investigated if people will cheat in the presence of an autonomous robot compared to being alone. They found a decrease in cheating behaviours in the presence of an autonomous robot compared to being alone.

In summary, several limitations exist in the current work, such as the passive role of the robot, use of artificial tasks and environment, and small sample size of the participants. Through this work, we attempt to address these limitations by developing a practical task and conducting a study with adults monitored by an autonomous robot invigilator.

### 2.2 Virtual *Versus* Physical Embodiment

The comparison of virtual and physical robot embodiment is not a novel phenomenon in HRI (Lee et al., 2006; Wainer et al., 2007; Bainbridge et al., 2008; Admoni and Scassellati, 2017). Several studies have shown that participants mostly enjoy interaction with a physical robot and perceive it as more watchful than a remote robot (teleoperated) or a virtual robot (Wainer et al., 2006). Physical robots could provide feedback through physical motions such as tilting forward or backward or moving their camera as if nodding a head.

A physical robot has disadvantages, such as added hardware and the need for maintenance and installation (Belpaeme et al., 2018). There are also compelling advantages for a robot compared to virtual agents, such as the ability of robots to teach a subject that requires physical engagement and pupils exhibiting better social behaviour for learning while engaging with robots. Better social behaviour is linked to higher engagement and more compliance with requests, even if they are difficult. Moreover, pupils indicate better learning gains upon interacting with a robot (Belpaeme et al., 2018).

In the context of the role of an invigilator, we found no studies that investigated the difference in people’s perception of a physical robot and a virtual agent and their role in preventing indiscipline or cheating. The closest work is by Mol et al. (2020), which investigated the effect of the presence of a virtual observer on cheating behaviour by enabling the virtual observer to stare at the participant in a virtual reality environment. However, the virtual observer played a passive role and did not detect signs attributed to indiscipline or cheating. Mol et al. did not undertake a comparison with a physically present robot.

### 2.3 Ethics and Exam Invigilation

Ethical integrity is essential in the context of exam invigilation. Both examinee and examiner need to view an invigilator as having moral authority and integrity. With the COVID-19 pandemic in the world, we may see an overhaul change in how exam invigilation happens in the future. For instance, a robot invigilator can be used for online and in-person exams in virtual, in-person, or teleoperated roles. However, this raises legal and ethical concerns regarding protecting a student’s privacy and safety (Colonna, 2021). The concerns may include 1) accurate selection of behaviours that can be categorised as cheating or indiscipline in an exam setup, 2) data security when relying on biometric data (face), especially when relying on public networks, and 3) discrimination (Colonna, 2021). The High-Level Expert Group on Artificial Intelligence has released an ethics guideline. The guidelines consider the following categories: respecting a person’s autonomy, preventing harm, being fair, and, finally, being understandable (Smuha, 2019).

The above provides a guideline for developing a more accurate, fair, and transparent robot invigilator. For this study, we attempted to develop a robot invigilator that follows the guidelines. In the context of the empirical investigation, it is significant to account for user perception of the developed systems. In particular, the amount of trust bestowed upon the invigilator and its perceived ethics and morals in the eyes of the examinee are important measurements that cannot be ignored. Hence, we used the multi-dimensional measure of trust (MDMT) questionnaires (Malle and Ullman, 2021) to measure the subjective ratings of invigilators’ performance and moral trust.

## 3 System Description

**FIGURE 1:**
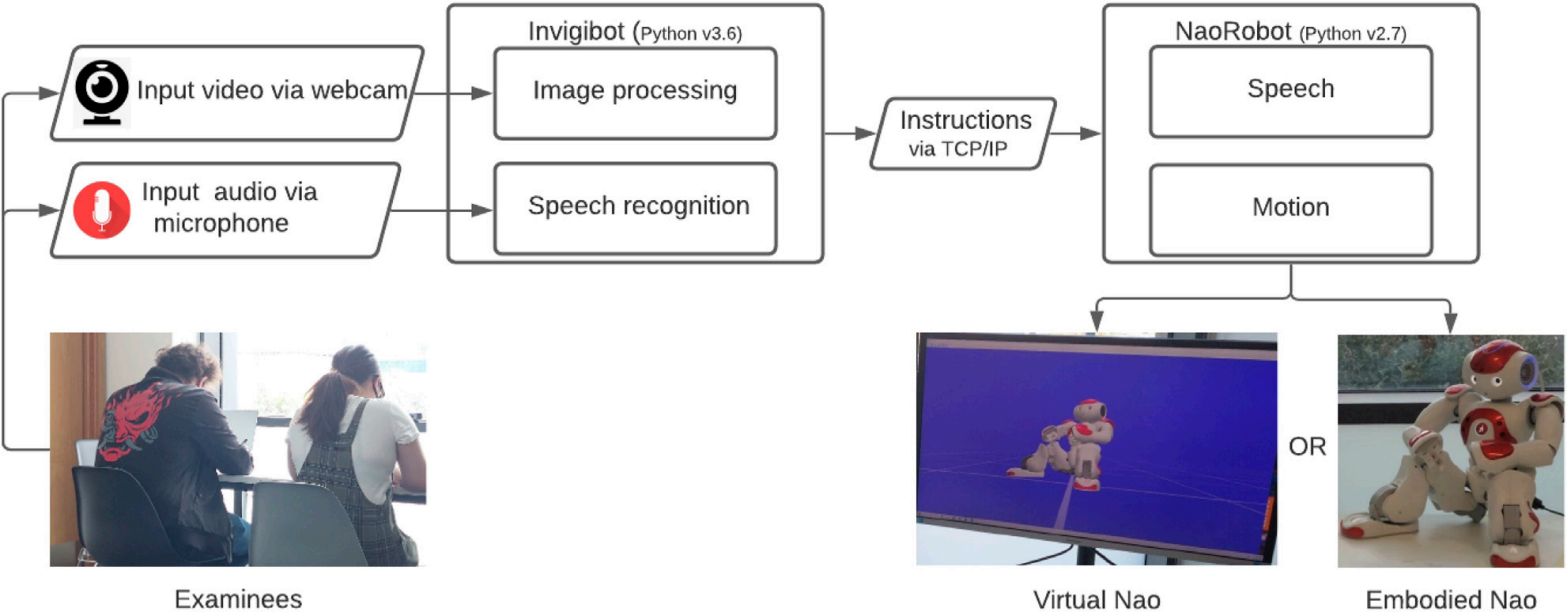
Robot invigilator system. Left: two examinee participants performing the task in the experiment. Right: the virtual Nao robot in Choregraphe software (left) and physical Nao robot (right) as used in the experiment.

The system shown in Figure 1 was implemented on a robot (physically or virtually present) to monitor two examinees in an exam setting. We encourage the HRI community to reuse our code to promote the reusability and reproducibility of the results.^1^ We understand that a real examination setup presents a greater challenge and involves monitoring several examinees. However, we demonstrate a proof of concept that can be modified for more individuals in the future. The system enables the robot to verbally warn the examinees in the two cases: 1) when either examinee looks towards each other or their exam sheets and 2) when any of the examinees engages in verbal interaction(s).

### 3.1 Components

The following components are required to execute the system: a Nao robot (real or virtual), a microphone, a camera, and a laptop for running the code. The camera is used to monitor the examinees’ head pose directions, and the microphone is used to listen to their speech.

### 3.2 Modules

We developed two modules: 1) the Invigibot module was used to monitor the examinees by analysing videos in real time and 2) the Nao robot module was used to control the robot’s speech and animation. The video and voice recordings were processed in the Invigibot module and used to trigger an animated verbal reaction from the Nao robot.

### 3.3 Invigibot

#### 3.3.1 Head Pose Tracking

**FIGURE 2:**
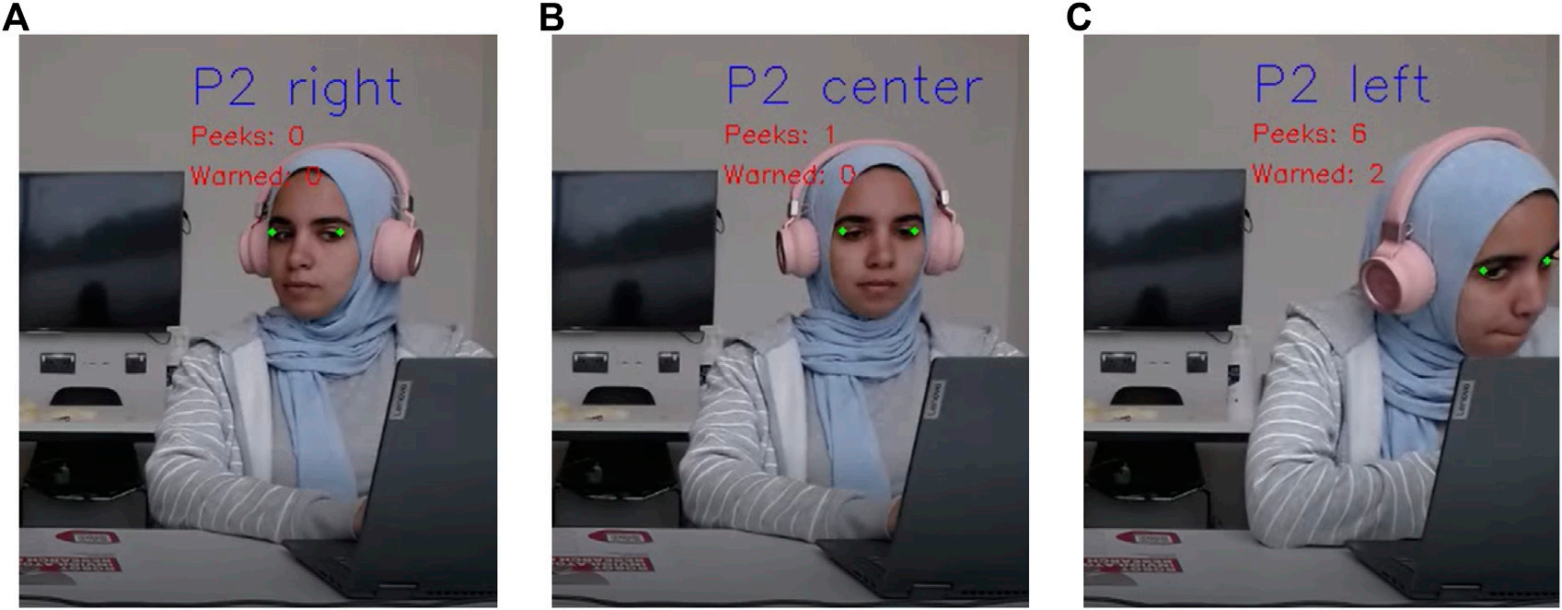
Head pose directions: directions **(A–C)** are from the point of view of the person in the photo. The counters “peeks” and “warned” appear in the recorded video for analysis purposes.

We implemented a head pose tracking method to detect if an examinee was looking at the exam sheet of the other examinee. We used the MediaPipe (MP) library to estimate the head pose (Canu, 2021). Head pose tracking is the deduction of a head’s orientation from a photo or a video (Murphy-Chutorian and Trivedi, 2008). The simplest methods can determine whether the head is posed in one of a discrete number of poses such as front, right, or left, as shown in Figure 2.

We used the MP library’s 468 facial landmark points to track head pose direction. First, we established that we need to know if an examinee is looking towards the centre, right, or left because we only care if they are looking towards each other or at their exam sheets. Therefore, we ignored if the examinees were looking up or down. Therefore, we extracted two points for each person. In the library, points 247 and 467 represented the outer corners of the eyes, as shown in Figure 2 in green. For each point, we took the x, y, and z coordinates. The x and y coordinates help us draw the dots on the image to ensure we track the correct area. The z coordinate helps detect the head pose direction. If point 247 is z_1_ and point 467 is z_2_, then we can estimate head pose direction by the following equation:

$$\Delta z=z_{2}–z_{1}$$

**FIGURE 3:**
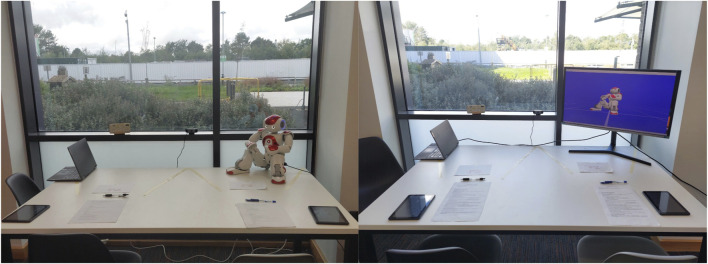
The setup was consistent for both physical robot and virtual robot conditions; only the type of invigilator is changed.

We do not get the direction by just calculating Δz. We need to determine the range within which an examinee can move their head and beyond which they look left or right. Hence, two strips of tape are placed in a V-shape on the table in front of each examinee (Figure 3) to show the allowed range of motion. This range is measured by asking the examinee to swivel their head from one strip of tape to the other and back for 5 s. Meanwhile, a video is captured, and the maximum and minimum Δz are recorded. When the examinees are asked to start their exam, they are also recorded and their Δz is constantly calculated. This gives us Δz_min_ and Δz_max_. We deduce the head pose direction using the below simple rules:

$$\Delta z<\Delta z_{\min};\text{ direction: right}$$

$$\Delta z>\Delta z_{\max};\text{ direction: left}$$

$$\Delta z_{\min}<\Delta z<\Delta z_{\max};\text{ direction: center}$$

Since the setup in this study was meant for only two examinees, the calibration and direction deduction process were done twice, once for each participant. The video recording showed two individuals. Hence, the obtained image is split in half. One half is participant 1 (P1), and the other is participant 2 (P2). Each participant had a counter for the number of peeks and the number of warnings they received. We know that P1 might be cheating if they look left, and P2 might be cheating if they look right. Therefore, these are the actionable behaviours for which they could receive a warning. In Figure 2, we show the Invigibot module in execution. We can see that the module accurately detects the direction of the participant (right, centre, or left).

When participants displayed actionable behaviours, they did not instantly receive a warning. Instead, the frame counter was initiated. Once the number of frames added up to one second, the peek counter was incremented by 1. The participant received a warning once the peek counter added up to 3. It also resulted in an increment to the warning counter. This was done to ensure an accurate selection of behaviours that can be categorised as cheating or indiscipline in an exam setup, hence ensuring integrity (Colonna, 2021). Furthermore, to give the invigilator a more human-like nature, as the warnings increased, the warning statement changed, so the invigilator was less monotonous and more human-like. The following are the three warning statements, where # is the participant number:

1. Participant #, please look at your paper.
2. Participant #, no peeking.
3. Participant #, I have warned you several times already.

We encountered problems detecting facial landmarks for two individuals in an image instead of one. The MP library detects several faces in one frame. However, when the number of faces was adjusted to two, landmarks for both participants were detected accurately, but the order by which they were detected was not constant. In a matter of seconds, z_1_ would refer to P1 and then to P2 and back. This, of course, created problems in detecting actionable behaviours accurately, as looking left is actionable for P1 but not for P2. Hence, we reverted to splitting the image in two and detecting only one face per image. This gave a good approximation for Δz.

#### 3.3.2 Listening

The other actionable behaviour is for a participant to talk during the exam. We detected talking through speech recognition. A session was voice recorded every 10 s. If the recording contained verbal responses, then we provided a warning to the participants. In efforts to give the invigilator more human features, the warning statement was selected randomly from the following:

• I can hear you; please be quiet.
• Talking is not allowed.
• No chit chat.

The warnings were not specific to one participant for the peeking. As we were using one microphone, we did not specify the source of the speech. Speech recognition worked accurately and almost always detected the presence of speech correctly. It did not pick up on writing, flipping papers, or clicking pens as noise. On the flip side, it sometimes seemed delayed due to the 10 s listening period. Moreover, participants could sometimes get away with speaking if they whispered in a low tone.

### 3.4 Nao Robot

The Nao robot, whether virtual or physical, was programmed to speak or move its head facing towards the concerned participant on each actionable behaviour. A TCP/IP server was implemented to enable communication between the Nao robot and the Invigibot modules to respond to the actionable behaviours.

## 4 Study Design

We tested the following hypotheses (H):

H1: **p**articipants will rate the physically present robot invigilator higher than the virtual robot invigilator in regard to the quality of invigilation (H1a), perceived intelligence (H1b), animacy (H1c), trust (H1d), and ethical integrity (H1e).

H2: **p**articipants will prefer the physically present robot invigilator over the virtual robot invigilator.

H3: **p**articipants will display more disciplinary behaviours (talking or peeking) in the exam setting when the invigilator is virtual.

H4: **p**articipants will display more cheating in the case of the virtual robot invigilator.

H5: **p**articipants’ speaking and peeking behaviours will decrease after the physically present robot invigilator’s warnings **(**H5a**)** and

will continue after the virtual robot invigilator’s warnings **(**H5b**)**.

To test our hypotheses, we conducted two different studies. In study one, individuals participated virtually by filling in an online questionnaire. The questionnaire showed two videos of the exam setup (virtual and physical) and then asked the participant to rate the invigilator shown in the video.

For study two, we created an exam-like setup, as shown in Figure 3, and asked participants to complete an exam task once while monitored by the physical Nao robot and again while monitored by the virtual Nao. Both studies (one online and the other in-person) were a within-subject design with two conditions: physical or virtual invigilator robots, which are shown in Figure 1. The choice of two conditions for the study was grounded in the previous work that compared the physical and virtual variants of the robots (Thellman et al., 2016; Vasco et al., 2019).

Nao robot has been designed and built by Aldebaran Robotics. It is a humanoid robot 58 cm in height. The Nao robot sits in a crouching position on the table and is connected to the laptop *via* an Ethernet cable. The laptop’s built-in microphone is used to catch audio, and, later, text-to-speech (TTS) is used for the robot to utter the response.

Virtual Nao was provided in the Choregraphe software provided by Aldebaran Robotics. Note that the virtual simulation of the Nao robot does not have a TTS feature but can play audio. Therefore, we recorded the required statements from the physical robot as WAV files and played them during the interaction. The rest of the animation and mechanism were the same as those for the physical Nao robot.

### 4.1 Ethics

For ethical integrity, an application was submitted to the university ethics board. The application was approved following a review process. The online study recruited participants through email, Facebook, LinkedIn, or WhatsApp groups. Participants were not necessarily related to the university. Hence, people from a wider demographic in terms of age participated in this study.

For the in-person study, we created a poster that informed people to participate in the study. Participants needed to 1) have an agreement to perform the study in pairs, 2) have a basic understanding of programming, and 3) be capable of completing two quizzes while being monitored by the robot. Participants were rewarded a maximum of *£*5 Amazon vouchers for their participation. Before beginning, all participants provided consent to being video recorded.

### 4.2 Study One (Online Study)

#### 4.2.1 Task

We recorded two 30 s short video clips where the authors acted as examinees and deliberately performed behaviours such as peeking and talking to show behaviours regarded as inappropriate in an exam setup. Both videos presented similar behaviours depicted by the examinees and, simultaneously, either the physically present Nao or the virtual Nao invigilator tried to prompt the examinees to focus on their exam papers.

Two questionnaires were created where the only difference was the order in which the videos appeared. The questionnaire asked for participants’ demographics and whether they had interacted with a robot before. Later, it presented the interaction video of either the physical or virtual invigilator and then asked the participant to rate the invigilator. Participants were randomly assigned to see either the virtual Nao invigilator video or the physical Nao invigilator video in a counterbalanced fashion. In summary, 41 watched the physical invigilator first, and 35 watched the virtual invigilator first.

#### 4.2.2 Participants

We recruited 76 participants (37 males, 38 females, 1 non-binary). Participants’ ages ranged in the following categories: 27 aged 18–25, 37 aged 25–35, 4 aged 35–45, 5 aged 45–55, and 3 aged 55–65. Twenty-four participants had experience in interacting with a robot.

#### 4.2.3 Procedure

Participants completed the following steps:

1. Participants watched the video of either the physical or virtual invigilator monitoring the examinees.
2. Participants completed the questionnaire to rate the invigilator.
3. Participants repeated steps 1 and 2 for the other invigilator type.

#### 4.2.4 Measurement

The study has the following measurements: i) quality of invigilation, ii) animacy, iii) perceived intelligence, iv) capacity trust, v) moral trust, and vi) preference of the invigilator.

To measure the quality of invigilation (see Table 1), we asked participants to rate the following statements on a Likert-like scale of 1–5: “the invigilator was clear,” “The invigilator warnings were justified,” and “I would feel monitored by the invigilator.” We computed the mean of the three ratings to estimate the quality of interaction. We conducted a Cronbach’s Alpha reliability test for the quality of invigilation. The two-part (physical vs. virtual invigilator) questionnaire was filled by 76 participants. The three-item quality of interaction subscale had the following values for the physical robot invigilator (*α* = 0.69) and virtual robot invigilator (*α* = 0.39), respectively.

**TABLE 1:** List of questions for quality of invigilation—study one.

Questions
The invigilator was clear
The invigilator warnings were justified
I would feel monitored by the invigilator?

To measure animacy and perceived intelligence, we use the items available in the Godspeed questionnaire series (Bartneck et al., 2009). These items include responsive, life-like, competent, and intelligent.

To measure capacity trust and moral trust, we use the MDMT questionnaire developed by Malle and Ullman (2021). The MDMT is a Likert scale to rate eight elements relevant to capacity and moral trust. The eight elements were averaged to measure capacity and moral trust. MDMT is a reliable scale for measuring humans’ trust in robots Ullman and Malle (2019) and has been widely used in HRI studies Chita-Tegmark et al. (2021).

We also asked the participants to give their preference for the embodiment of the invigilator. Lastly, we asked them to explain why that type was preferred to get qualitative impressions. We used qualitative data to discuss the findings obtained from the study.

#### 4.2.5 Results

Through this study, we tested H1 and H2**,** respectively. A Kolmogorov–Smirnov (KS) test indicated that the data were not normally distributed (*p* < 0.05). Hence, to test H1, we conducted a Wilcoxon signed-rank test to examine the differences in the participant’s ratings for quality of invigilation (H1a), perceived intelligence (H1b), animacy (H1c), trust (H1d), and ethical integrity (H1e) according to the conditions. The analysis indicates that participants rated the quality of invigilation for the physical robot invigilators significantly higher than the virtual robot invigilator Z = −2.7017, *p* < 0.01. We did not observe significant difference between conditions for the rest of the measurements. The mean and standard deviation (SD) values for all measurements can be seen in Table 2.

**TABLE 2:** Measurements of study one: mean and SD.

Measurements	Physical invigilator		Virtual invigilator	
	Mean	SD	Mean	SD
Quality of invigilation	3.99	0.78	3.75	0.87
Animacy	3.16	0.95	3.28	0.96
Intelligence	3.42	0.95	3.33	1.00
Trust	3.25	0.95	3.34	0.93
Ethics	3.63	1.09	3.59	1.10

To test H2, we conducted a Chi-square analysis on the observed frequencies for the preferred embodiment of the invigilator. We found a statistically significant preference for the physical robot invigilator (68.42% for physical vs. 31.58% for virtual), *χ* ^2^ (2, 76) = 10.31, *p* < 0.002.

### 4.3 Study Two (In-Person Study)

#### 4.3.1 Task

Participants were asked to complete two short quizzes. Each consisted of 10 multiple choice questions on programming topics such as data structures, efficiency, and errors. We ensured that both quizzes had a similar level of difficulty. The rationale for choosing a programming task was to ensure that the presented task was real and challenging. Past work has considered tasks that can be argued as easy, artificial, and not challenging.

In order to induce indiscipline or cheating behaviour, the answers to each participant’s quiz were shared with the other participant (Mubin et al., 2020). Furthermore, the reward (maximum *£*5) depended on the number of correct answers to the quiz questions. We used this strategy because it has been utilised in the past literature (Petisca et al., 2020). Furthermore, we felt that providing financial incentives may induce cheating behaviours as it is one of the reasons individuals involve themselves in cheating behaviours (Amigud and Lancaster, 2019).

#### 4.3.2 Participants

We recruited 26 participants (21 males and 5 females). Participants’ age ranged in the following manner: 14 aged 18–25, 10 aged 25–35, and 2 aged 35–45. Eleven participants had an experience of interacting with a robot. All participants were either university students or staff.

#### 4.3.3 Procedure

The study was conducted with two participants at a time. Each time the below steps were followed to complete the study:

1. Participants were welcomed and asked to take a seat. Then, they were provided information sheets and requested to read and sign the consent forms.
2. Participants filled a pre-questionnaire using a tablet and were briefed on the process.
3. Participant 1 was given test version A, whereas participant 2 was given test version B. Both participants were informed that they had different versions and that each had the answers to the other person’s test at the back of their test. They were also informed that their Amazon voucher’s worth increases as they get more correct answers, with a maximum of *£*5.
4. The experimenter later executed the code to begin the interaction. The robot (physical or virtual) explained to the participants that it would be their invigilator and that they had 5 minutes to complete the quiz. Later, the invigilator asked the participants to swivel their heads between the tape lines in front of them to calibrate their allowed range of head motion or Δz. Finally, the invigilator asked participants to start solving their tasks.
5. Once the robot said, “time is up,” the quizzes were collected and each participant filled the post-questionnaire.
6. The invigilator type was exchanged for the other type. Each appointment was alternately started with the robot invigilator and then the virtual invigilator, so the results were not affected by the order of the invigilators. The participants were given opposite versions of the tests at that time, so participant 1 got version B and participant 2 got version A. They still had the answers to the other person’s test. Steps 4 and 5 were repeated for the new invigilator. The second post-questionnaire asked participants to indicate their preferred type of invigilator.
7. Participants were thanked for their time and asked for further comments.

For each interaction, a video was recorded and saved in a file with the name “Appointment number + test number + invigilator type.”

#### 4.3.4 Setup and Materials

The study had three different questionnaires: i) a pre-questionnaire that asked about the participant’s gender and age and whether they have had previous experience with a robot, ii) a questionnaire that asked participants to rate their first interaction with the invigilator, and iii) a questionnaire that asked participants to rate the second type of invigilator, choose their preferred type of invigilator, and give a reason for their choice.

The setup can be seen in Figure 3. It consisted of an in-built laptop’s microphone and a camera placed in front of a table with two seats for each participant. A laptop was placed in the left corner to enable the researchers to begin the experiment. The invigilator, either robot or virtual agent, was placed in the right corner, as shown in Figure 3. After executing the program on the robot, the experimenter sat next to the door of the room with their head in the opposite direction to avoid the impact of any confounding factor(s). All factors (size of the Nao (in-person or on-screen), behaviours, and proximity) were carefully, meticulously, and consistently maintained in both conditions.

#### 4.3.5 Measurement

This study has measurements similar to the first (online) study: i) quality of invigilation (see Table 3), ii) animacy, iii) perceived intelligence, iv) capacity trust, v) moral trust, and vi) preference of the invigilator.

**TABLE 3:** List of questions for quality of invigilation—study two.

Questions
The invigilator was clear
The invigilator warnings were justified
I felt monitored by the invigilator

We conducted a Cronbach’s Alpha reliability test for the quality of invigilation. The two-part (physical vs. virtual invigilator) questionnaire was filled by 26 participants. The three-item quality of interaction subscale had the following values for the physical robot invigilator (*α* = 0.66) and virtual robot invigilator (*α* = 0.70), respectively. For other metrics, we did not run the reliability checks because these subjective metrics have been reliably tested and used in many studies (Bartneck et al., 2009; Ullman and Malle, 2019).

Further behavioural measures are noted: i) the number of peeks, ii) the times a participant speaks, iii) the times they are warned, iv) whether or not cheating is attempted, and v) test scores. This helps us check for a correlation between the type of invigilator and indiscipline or cheating behaviours displayed. Measures i, ii, and iii are counted frequencies. Cheating attempts are a true or false measure (0 or 1). Finally, tests are marked out of 10, such that 10 correct answers give a full mark of 10.

#### 4.3.6 Results

Through this study, we test hypotheses H1, H2, H3, H4, and H5, respectively. A KS test indicated that the data were normally distributed (*p* > 0.05). Hence, to test hypothesis H1, we conducted an independent-samples *t*-test to analyse the difference in ratings (physical vs. virtual) for quality of invigilation (H1a), perceived intelligence (H1b), animacy (H1c), trust (H1d), and ethical integrity (H1e). The analysis did not indicate significant differences in the participants’ ratings of the measures. The only exception was a slightly significant difference in animacy, where the robot invigilator was rated higher than the virtual invigilator (*p* = 0.09).

To test H2, we conducted the Chi-square analysis for the preferred type of invigilator chosen by participants. However, no statistically significant difference is found in terms of preference for the physical robot invigilator (65.38% for physical vs. 34.61% for virtual), *χ* ^2^ (2, 26) = 2.46, *p* = 0.11.

In order o test H3 and H4, a paired *t*-test was conducted to determine if a statistically significant difference exists in participant’s behaviour metrics (peeking, speaking, or cheating) for the interaction session with the robot *versus* the virtual agent. The means of the 26 participants’ speaking behaviour from the two conditions are found to be significantly different, *t* (27) = –2.39, *p* < 0.03 (two-tailed). It suggests that participants’ speaking behaviour was significantly higher in the virtually present agent condition. No significant difference in peeking or cheating behaviours is found. The mean and SD can be seen in Table 4.

**TABLE 4:** Measurements of study two: mean and SD.

Measurements	Physical invigilator		Virtual invigilator	
	Mean	SD	Mean	SD
Quality of invigilation	3.65	0.89	3.53	0.85
Animacy	3.15	1.14	2.88	1.34
Intelligence	3.27	0.93	3.13	1.11
Trust	3.40	0.83	3.38	0.82
Ethics	3.63	1.03	3.64	1.02
Peeking	0.42	1.39	0.5	1.07
Speaking	0.73	1.82	1.54	2.60
Warnings	1.69	3.37	1.96	3.43
Cheating attempted	0.12	0.33	0.19	0.40
Test scores	5.69	2.54	5.38	1.83

To test H5a, we conducted a Pearson correlation coefficient to assess the relationship between the physically present robot invigilator’s warnings, participants’ peeking, and speaking behaviours. There was a moderate positive correlation between the robot invigilator’s warning and participants’ peeking behaviour *r* (25) = 0.35, *p* = 0.001. We did not see a significant relationship between the invigilator’s warning and participants’ speaking behaviour.

To test H5b, we conducted a Pearson correlation coefficient to assess the relationship between the virtual robot invigilator’s warnings, participants’ peeking, and speaking behaviours. There was a strong positive correlation between the virtual robot invigilator’s warning and participants’ speaking behaviour *r* (25) = 0.61, *p* < 0.000. We did not see a significant relationship between the invigilator’s warning and participants’ peeking behaviour.

## 5 Discussion

H1a predicted that the quality of invigilation for the physically present robot invigilator would be rated higher than the virtual invigilator. The quality of invigilation included three qualities: clarity, feeling monitored, and justified warnings. The findings of the online study (study one) confirmed H1a. However, it was not verified based on the findings of the in-person evaluation (Study two). This is a thought-provoking finding and calls for the discourse in the HRI community in regard to treating the results collected from video-based evaluations rather carefully. In the context of this study, we understand that both physical and virtual robot invigilators were similar in actions. In our case, this effect was more pronounced in the physical interaction with both embodiments. However, the videos used for virtual and physical were similar. We witness that the in-person and virtual experience does impact the findings (Itani and Hollebeek, 2021).

H1b, H1c, H1d, and H1e predicted higher ratings for the physically present invigilator with regard to intelligence, animacy, trust, and ethical integrity, respectively. In general, no statistically significant effects between the ratings provided for the physical and the virtual invigilators were seen. Therefore, all four hypotheses were rejected. This finding contradicts the existing work that suggests the physical presence of the robot makes participants regard the robot as more respectful and trustworthy (Bainbridge et al., 2008). We did not find the case in the invigilation or monitoring role of the robot. We speculate that the context of invigilation impacted the participants’ ratings concerning the fundamental question on the benefits of the physical robot to the virtual agent. We appreciate that participants were not willing to cheat. Often, they remain focused on their task. It suggests that participants, being university staff members and students, exercised the high moral ground and appreciated the authority of the robot in the role of an invigilator in the in-person study. For the online study, we used the university mailing lists and understood that the same effect might have been pronounced in the overall findings.

H2 predicted that participants would prefer the physically present invigilator over the virtual one. The online study (study one) confirms H2 with a profoundly significant difference in favour of the physical robot. Even though the in-person study (study two) results show that around 65% of the participants preferred the physical robot, the Chi-Square test did not show a significant difference. The percentage of participants’ preference for the physical invigilator in the online study was around 68%, yet the number of participants was 76. We discuss these findings through an eye of qualitative insights. Participants who preferred the physical invigilator in the in-person study commented, “closer to reality,” “felt livelier,” “more of a physical presence,” “felt monitored,” “can be humanized,” “more personal,” “looked cooler,” “the virtual agent doesn’t feel like it was there,” and “good interaction.” On the contrary, participants who preferred the virtual agent said, “less toy-like,” “size of the monitor made me more scared of cheating,” and “the robot is creepy.” All these insights are compelling and perhaps share one of the findings of Mubin et al. (2020). Mubin et al. suggested that the size of the robot and the cuteness of the robot make Nao have less authority and young participants did not take Nao seriously in the role of an invigilator. Perhaps this could also be the reason for this finding.

H3 predicted that participants would display more indiscipline behaviour while monitored by the virtual invigilator. We investigated this hypothesis in the in-person study (study two). The in-person study showed that the number of peeks made by participants did not differ significantly across the different types of invigilators. However, participants engaged in verbal dialogue much more while monitored by a virtual invigilator. Hence, H3 was partially accepted. As participants were in pairs, we speculated that this effect was due to the familiarity or relationship between the two participants. However, we did not observe it to be the case. We randomly allocated participants in pairs. On some occasions, they knew each other. However, we noticed that all groups that spoke during the study were more talkative during the virtual agent condition. The more talkative behaviour can be linked to the depletion of perceived attention in the presence of the virtual robot invigilator. Studies have shown that it can lead to more cheating or indiscipline, as the presence of an invigilator and or their highly perceived attention among examinees is needed to overwhelm the examinees’ desire to not speak during an exam setting. (Gino et al., 2009; Mead et al., 2009; Pitesa et al., 2013). Another reason could be that participants may have felt they were not being watched in the presence of the virtual invigilator, so their sense of privacy and anonymity may not have raised concerns. This could have led to more talkative behaviours (Ayal et al., 2015).

H4 predicted that participants would attempt more cheating while monitored by the virtual invigilator. The results show rare occurrences of cheating across both types of invigilators. We did not observe a significant difference between the two conditions. Hence, H4 was rejected. This finding is similar to the one reported by Mubin et al. (2020). We appreciate that it is certainly challenging to mimic a real exam. Although we tried to maintain a challenging exam task and created a reward mechanism to entice cheating (Petisca et al., 2020), it was difficult to recreate the fear of failure and stress caused by a real exam (Küçüktepe, 2014; Fendler and Godbey, 2016). These factors affect a student’s propensity to cheat. Some participants did not engage in any disciplinary behaviour, so the invigilator never prompted them. As they had not experienced the invigilator warning them, they might not have provided an informed rating. One participant suggested that had they been given a scenario and implicitly asked to speak or try to cheat, they would have witnessed the invigilator’s warnings, which may have enabled them to rate both types more accurately. Another participant said they still felt cheating was wrong even though they knew it was an inconsequential test. It shows how personal beliefs and ethics affect the propensity to cheat regardless of the invigilator type. Another explanation can be attributed to the cultural background of the participants, as it has been stated as a factor influencing undergraduate students’ misconduct activity (Culwin, 2006; Wilkinson, 2009).

Finally and surprisingly, H5a and H5b predicted that there would be a decrease in participants’ peeking and speaking behaviour after the physically present robot invigilator’s warning, whereas participants’ peeking and speaking behaviour would continue or increase after the virtual robot invigilator’s warnings. We did not observe a significant decrease in participants’ peeking and speaking behaviour in the physically present robot invigilator condition. Hence, H5a was not accepted. On the contrary, we saw a significant increase in participants’ speaking behaviour with an increase in the virtual robot invigilator’s warnings. However, we did not find a relationship in regard to peeking behaviour. Hence, H5b was partially accepted. These findings are fascinating. However, we remain cautious regarding them. We observed during both conditions that, on very few occurrences (once for physical robot and twice for virtual robot), participants received unjustified peeking warnings. The system worked seamlessly to detect speaking behaviours. For instance, we observed that, in the physical robot invigilator condition, P1 looked at P2 when P1 asked for answers, P1 was warned to look to their paper, and P2 did not provide any answers. Both P1 and P2 laughed the first time and received another warning. In the virtual condition, participants also found more creative ways. For instance, one participant was peeking without moving their head and was also moving the answers’ paper towards the other participant. The results are indeed intriguing as they did highlight interesting and insightful behaviours. In summary, this research space still needs more exploration as limited studies have explored whether humans will comply with robots or agents (Mizumaru et al., 2019; Jois and Wagner, 2021; Petisca et al., 2022).

## 6 Conclusion and Future Work

We developed a system to detect cheating, which was deployed on a physical and a virtual Nao robot. We tested the system to check its applicability in the context of an exam where we recruited participants to complete two multiple-choice tests on the topic of programming, once while monitored by a physical invigilator and once while monitored by a virtual invigilator. We also conducted an online study where participants were shown clips of both types of invigilators and asked to rate them as if they were one of the examinees.

We investigated the effect of embodiment on cheating and disciplinary behaviours. We questioned whether participants regarded the invigilators differently in terms of quality of invigilation, trust, intelligence, animacy, and ethics. We found no significant difference between the physically present robot invigilator and the virtual robot invigilator in terms of trust, intelligence, and ethics. Moreover, there is no significant difference in the number of cheating attempts. The in-person study shows that participants were more likely to display undisciplined behaviour such as speaking while monitored by the virtual agent. In contrast, the online study shows a significant difference in the perceived quality of invigilation in favour of the embodied robot. Participants seemed to prefer the robot invigilator to the virtual agent as it was more present and life-like, according to their comments. Some preferred the robot because it was a novel experience.

In conclusion, physically present robots were preferred by participants over virtual agents even though they were both different embodiments of the same system. In terms of the implication of the presented work, we learn that we need to test robot invigilators in a more complex and real context to recreate the fear of failure and stress caused by a real exam. It could lead to achieving more subtle findings. Nonetheless, the findings are based on a large pool of participants and show that the physically present robots may be more useful. However, we understand that the development of the system design remains the key contributor. It may result in achieving excellent invigilation outcomes regardless of the embodiment.

In the future, we aim to deploy the system with four−eight participants at a time and intend to adjust the tracking method for head pose estimation. Moreover, we will adjust the listening feature for lower decibels and try to amplify whispers successfully. We will add microphones for each participant to enable us to provide more personalised warnings for speaking. Further, we aim to create a more complex and real context that is even more close to the exam-like setup.

## Data Availability

The raw data supporting the conclusion of this article will be made available by the authors without undue reservation.

## Footnotes

1
https://github.com/reemrafik/Robot-Invigilator.

## References

[B1] Admoni H. Scassellati B. (2017). Social Eye Gaze in Human-Robot Interaction: a Review. J. Human-Robot Interact. 6, 25–63. 10.5898/jhri.6.1.admoni

[B2] Ahmad M. I. Adeola E. S. Mubin O. (2021). “Towards the Applicability of the Social Robot in the Role of an Invigilator,” in Proceedings of the 9th International Conference on Human-Agent Interaction (New York, NY, USA: Association for Computing Machinery). 10.1145/3472307.3484665

[B3] Ahmad M. Mubin O. Orlando J. (2017). A Systematic Review of Adaptivity in Human-Robot Interaction. Multimodal Technol. Interact. 1, 14. 10.3390/mti1030014

[B4] Alves-Oliveira P. Sequeira P. Paiva A. (2016). “The Role that an Educational Robot Plays,” in 2016 25th IEEE International Symposium on Robot and Human Interactive Communication (RO-MAN). 10.1109/roman.2016.7745213

[B5] Amigud A. Lancaster T. (2019). 246 Reasons to Cheat: An Analysis of Students' Reasons for Seeking to Outsource Academic Work. Comput. Educ. 134, 98–107. 10.1016/j.compedu.2019.01.017

[B6] Anderman E. M. Murdock T. B.(Editors) (2007). Psychology of Academic Cheating. Burlington, MA: Elsevier Academic Press.

[B7] Ayal S. Gino F. Barkan R. Ariely D. (2015). Three Principles to REVISE People's Unethical Behavior. Perspect. Psychol. Sci. 10, 738–741. 10.1177/1745691615598512 26581728

[B8] Bainbridge W. A. Hart J. Kim E. S. Scassellati B. (2008). “The Effect of Presence on Human-Robot Interaction,” in RO-MAN 2008-The 17th IEEE International Symposium on Robot and Human Interactive Communication (IEEE), 701–706. 10.1109/roman.2008.4600749

[B9] Bartneck C. Croft E. Kulic D. Zoghbi S. (2009). Measurement Instruments for the Anthropomorphism Animacy Likeability Perceived Intelligence and Perceived Safety of Robots. Int. J. Soc. Robotics 1 (1), 71–81. 10.1007/s12369-008-0001-3

[B10] Belpaeme T. Kennedy J. Ramachandran A. Scassellati B. Tanaka F. (2018). Social Robots for Education: A Review. Sci. Robot. 3, eaat5954. 10.1126/scirobotics.aat5954 33141719

[B11] [Dataset] Canu S. (2021). Facial Landmarks Detection—With Opencv, Mediapipe and python - Pysource.

[B12] Chita-Tegmark M. Law T. Rabb N. Scheutz M. (2021). “Can You Trust Your Trust Measure?,” in Proceedings of the 2021 ACM/IEEE International Conference on Human-Robot Interaction (HRI ’21) (New York, NY: Association for Computing Machinery), 92–100. 10.1145/3434073.3444677

[B13] Colonna L. (2021). Legal Implications of Using Ai as an Exam Invigilator. Faculty of Law, Stockholm University Research Paper.

[B14] Council B. (2019). Annual Report and Accounts 2018-19, 106.

[B15] Council B. (2020). Annual Report and Accounts 2019-20, 78.

[B16] Culwin F. (2006). An Active Introduction to Academic Misconduct and the Measured Demographics of Misconduct. Assess. Eval. High. Educ. 31, 167–182. 10.1080/02602930500262478

[B17] Deng E. Mutlu B. Mataric M. (2019). “Embodiment in Socially Interactive Robots,” in Foundations and Trends® in Robotics Vol.7, 251–356. 10.1561/2300000056

[B18] Fendler R. J. Godbey J. M. (2016). Cheaters Should Never Win: Eliminating the Benefits of Cheating. J. Acad. Ethics 14, 71–85. 10.1007/s10805-015-9240-8

[B19] Gino F. Ayal S. Ariely D. (2009). Contagion and Differentiation in Unethical Behavior: The Effect of one Bad Apple on the Barrel. Psychol. Sci. 20, 393–398. 10.1111/j.1467-9280.2009.02306.x 19254236

[B20] Hoffman G. Forlizzi J. Ayal S. Steinfeld A. Antanitis J. Hochman G. (2015). “Robot Presence and Human Honesty: Experimental Evidence,” in 2015 10th ACM/IEEE International Conference on Human-Robot Interaction (HRI) (IEEE), 181–188.

[B21] Itani O. S. Hollebeek L. D. (2021). Light at the End of the Tunnel: Visitors’ Virtual Reality (Versus In-Person) Attraction Site Tour-Related Behavioral Intentions During and Post-Covid-19. Tour. Manag. 84, 104290. 10.1016/j.tourman.2021.104290 PMC9734089 36530603

[B22] Jois H. Wagner A. R. (2021). What Happens when Robots Punish? Evaluating Human Task Performance during Robot-Initiated Punishment. J. Hum.-Robot Interact. 10, 1–18. 10.1145/3472207

[B23] Küçüktepe S. E. (2014). College Students' Cheating Behaviors. Soc. Behav. Pers. 42, 101S–111S. 10.2224/sbp.2014.42.0.s101

[B24] Lee K. M. Jung Y. Kim J. Kim S. R. (2006). Are Physically Embodied Social Agents Better Than Disembodied Social Agents?: the Effects of Physical Embodiment, Tactile Interaction, and People's Loneliness in Human–Robot Interaction. Int. J. human-computer Stud. 64, 962–973. 10.1016/j.ijhcs.2006.05.002

[B25] Malle B. F. Ullman D. (2021). “A Multidimensional Conception and Measure of Human-Robot Trust,” in Trust in Human-Robot Interaction (Elsevier), 3–25. 10.1016/b978-0-12-819472-0.00001-0

[B26] Mead N. L. Baumeister R. F. Gino F. Schweitzer M. E. Ariely D. (2009). Too Tired to Tell the Truth: Self-Control Resource Depletion and Dishonesty. J. Exp. Soc. Psychol. 45, 594–597. 10.1016/j.jesp.2009.02.004 20047023 PMC2680601

[B27] Mizumaru K. Satake S. Kanda T. Ono T. (2019). “Stop Doing it! Approaching Strategy for a Robot to Admonish Pedestrians,” in 2019 14th ACM/IEEE International Conference on Human-Robot Interaction (HRI) (IEEE), 449–457. 10.1109/hri.2019.8673017

[B28] Mol J. M. van der Heijden E. C. Potters J. J. (2020). (Not) Alone in the World: Cheating in the Presence of a Virtual Observer. Exp. Econ. 23, 961–978.

[B29] Mubin O. Cappuccio M. Alnajjar F. Ahmad M. I. Shahid S. (2020). Can a Robot Invigilator Prevent Cheating? AI Soc 35, 981–989. 10.1007/s00146-020-00954-8

[B30] Mubin O. Stevens C. J. Shahid S. Al Mahmud A. Dong J.-J. (2013). A Review of the Applicability of Robots in Education. J. Technol. Educ. Learn. 1, 13. 10.2316/journal.209.2013.1.209-0015

[B31] Murphy-Chutorian E. Trivedi M. M. (2008). Head Pose Estimation in Computer Vision: A Survey. IEEE Trans. Pattern Anal. Mach. Intell. 31, 607–626. 10.1109/TPAMI.2008.106 19229078

[B32] Park E.-J. Park S. Jang I.-S. (2013). Academic Cheating Among Nursing Students. Nurse Educ. Today 33, 346–352. 10.1016/j.nedt.2012.12.015 23357719

[B33] Petisca S. Leite I. Paiva A. Esteves F. (2022). Human Dishonesty in the Presence of a Robot: The Effects of Situation Awareness. Int. J. Soc. Robotics. 10.1007/s12369-022-00864-3

[B34] Petisca S. Paiva A. Esteves F. (2020). “The Effect of a Robotic Agent on Dishonest Behavior,” in Proceedings of the 20th ACM International Conference on Intelligent Virtual Agents, 1–6. 10.1145/3383652.3423953

[B35] Pitesa M. Thau S. Pillutla M. M. (2013). Cognitive Control and Socially Desirable Behavior: The Role of Interpersonal Impact. Organ. Behav. Hum. Decis. Process. 122, 232–243. 10.1016/j.obhdp.2013.08.003

[B36] Rahim H. Rahiem M. D. H. (2012). The Use of Stories as Moral Education for Young Children. Int. J. Soc. Sci. Humanity 2, 454.

[B37] Rosanda V. Starcic A. I. (2019). “The Robot in the Classroom: a Review of a Robot Role,” in International Symposium on Emerging Technologies for Education (Springer), 347–357.

[B38] Smuha N. A. (2019). The Eu Approach to Ethics Guidelines for Trustworthy Artificial Intelligence. Comput. Law Rev. Int. 20, 97–106. 10.9785/cri-2019-200402

[B39] Thellman S. Silvervarg A. Gulz A. Ziemke T. (2016). “Physical vs. Virtual Agent Embodiment and Effects on Social Interaction,” in International conference on intelligent virtual agents (Springer), 412–415. 10.1007/978-3-319-47665-0_44

[B40] Ullman D. Malle B. F. (2019). “Measuring Gains and Losses in Human-Robot Trust: Evidence for Differentiable Components of Trust,” in 2019 14th ACM/IEEE International Conference on Human-Robot Interaction (HRI) (IEEE), 618–619. 10.1109/hri.2019.8673154

[B41] Vasco V. Willemse C. Chevalier P. De Tommaso D. Gower V. Gramatica F. (2019). “Train with Me: a Study Comparing a Socially Assistive Robot and a Virtual Agent for a Rehabilitation Task,” in International Conference on Social Robotics (Springer), 453–463. 10.1007/978-3-030-35888-4_42

[B42] Wainer J. Feil-Seifer D. J. Shell D. A. Mataric M. J. (2007). “Embodiment and Human-Robot Interaction: A Task-Based Perspective,” in RO-MAN 2007-The 16th IEEE International Symposium on Robot and Human Interactive Communication (IEEE), 872–877. 10.1109/roman.2007.4415207

[B43] Wainer J. Feil-Seifer D. Shell D. Mataric M. (2006). “The Role of Physical Embodiment in Human-Robot Interaction,” in ROMAN 2006 - The 15th IEEE International Symposium on Robot and Human Interactive Communication, 117122. 10.1109/ROMAN.2006.314404

[B44] Wilkinson J. (2009). Staff and Student Perceptions of Plagiarism and Cheating. Int. J. Teach. Learn. High. Educ. 20, 98–105.

